# Pharmacological therapies for type 2 diabetes: future approaches

**DOI:** 10.1007/s00125-025-06581-6

**Published:** 2025-10-31

**Authors:** Clifford J. Bailey

**Affiliations:** Health and Life Sciences, Aston University, Birmingham, UK

**Keywords:** Amylin, Glucagon, Glucose lowering, Incretin, Insulin action, Insulin secretion, Review, Therapies, Type 2 diabetes

## Abstract

Despite the availability of at least nine differently acting classes of glucose-lowering agents, many people with type 2 diabetes do not achieve or maintain sufficiently tight glycaemic control to avoid the complications of chronic hyperglycaemia. This narrative review examines the prospects for future non-insulin agents and therapeutic approaches in early development that aim to improve glycaemic control in type 2 diabetes. Such therapies will ideally enhance glucose lowering through existing mechanisms or by targeting different aspects of disease pathophysiology. They will avoid overt hypoglycaemia and facilitate weight control and be convenient to use, have minimal adverse effects, provide benefits against common comorbidities and have a commendable overall safety profile. Particularly promising therapies in development are the co-agonist and multi-agonist incretin-based and amylin-based synthetic peptides that improve glycaemic control and body weight regulation. Initial studies suggest that such therapies can improve insulin secretion, assist pancreatic beta cell preservation and enhance insulin-mediated glucose metabolism while reducing glucagon secretion and risk of fatty liver disease. Antibodies and small molecules that interact with incretin targets are also being developed, as well as agents to modulate mitochondrial function, fatty acid receptors and receptors for selected gastrointestinal and adipocyte peptides that affect appetite or pathways of nutrient metabolism. Multiomics, miRNAs, gene-editing technologies and epigenetic targets have received considerable attention but have yet to deliver usable therapies. Directing therapeutic agents to specific organs or tissues and avoiding unwanted off-target effects continue to challenge the application of laboratory innovations into viable clinical agents. However, recent successes with weight-lowering incretin-based medicines have raised expectations for pharmaceutical pipelines to transform the management of type 2 diabetes.

**Graphical Abstract:**

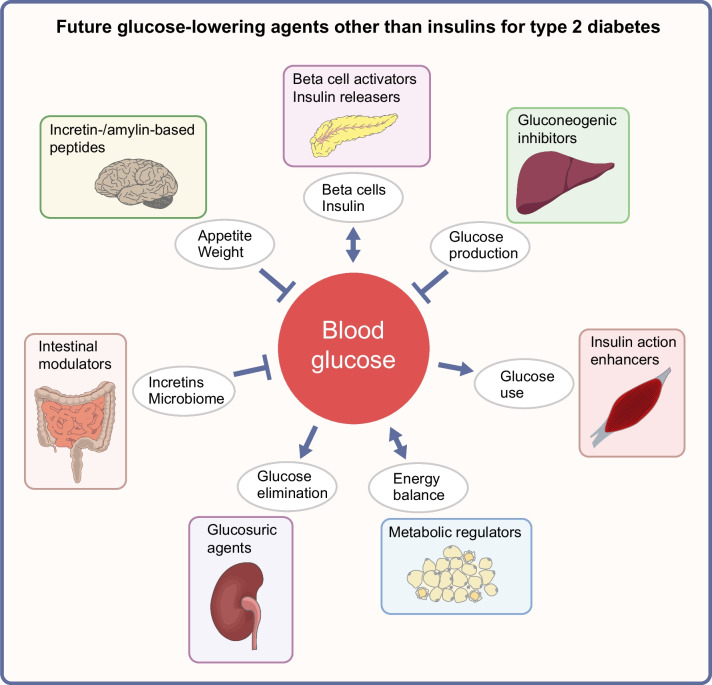

**Supplementary Information:**

The online version contains peer-reviewed but unedited supplementary material including a slide of the figure for download available at 10.1007/s00125-025-06581-6.

## Introduction

There are presently nine classes of glucose-lowering agents used in the treatment of type 2 diabetes in Europe and several more are available in other regions (Table 1) [1, 2]. Lifestyle changes (principally diet, exercise and behavioural interventions) underpin all treatment algorithms. Non-insulin pharmacological agents use different glucose-lowering mechanisms and can be used as monotherapy or in combination, while insulin provides the default medicine for the most advanced stages of this progressive disease [2–4]. Different agents can address different aspects of disease pathophysiology, intervene against complications and comorbidities, and act in parallel to facilitate a personalised therapeutic approach. However, normal glucose homeostasis is seldom reinstated. In surveys, >40% of individuals with type 2 diabetes typically fail to achieve or maintain recommended targets for HbA_1c_ and almost all eventually develop a micro- and/or macrovascular complication associated with inadequate metabolic control (electronic supplementary material (ESM) Appendix 1). These shortcomings highlight the need for further, differently acting and more effective therapies that are complementary to and compatible with existing agents and that have acceptable long-term safety profiles (ESM Appendix 2).

**Table 1:** Key features of the main glucose-lowering agents used in the management of type 2 diabetes

Class with examples^b^	Mode of action	(a) Glucose-lowering efficacy^a^ (b) Hypo risk^a^ (c) Weight effects^a^	(a) Cardiorenal effects (b) Cautions and limitations (c) Additional effects or comments
Oral			
Biguanide (metformin [IR, SR/XR formulations])	Counter insulin resistance: reduce hepatic glucose output, increase splanchnic glucose turnover, increase insulin-mediated glucose metabolism	(a) Efficacy high (b) Hypo risk low (c) Weight neutral	(a) Decrease CV mortality rate, increase survival rate, no evident effect on renal function but adequate renal function required for drug clearance (e.g. ≥30 ml/min per 1.73 m^2^) (b) Check renal function. Interrupt if using contrast media. Avoid in renal or liver impairment, sepsis, hypoxaemia, history of lactic acidosis or alcohol abuse. Rare risk of lactic acidosis. Check vitamin B_12_ in long-term use (c) Possible reduced risk of some cancers
Sulfonylureas (glibenclamide, gliclazide, gliclazide MR, glimepiride, glipizide, tolbutamide)	Stimulate insulin secretion (typical duration 6–24 h)	(a) Efficacy high (b) Hypo risk moderate (c) Weight gain	(a) Clinically significant cardiorenal effects not established (b) Avoid in renal or liver impairment depending on elimination route of agent. Self-monitor blood glucose if driving or operating machinery (c) Glucose-lowering efficacy declines with advancing beta cell dysfunction
Meglitinides (nateglinide, repaglinide)	Stimulate insulin secretion (rapid- and short-acting duration <6 h)	(a) Efficacy intermediate (b) Hypo risk moderate (c) Weight gain	(a) Clinically significant cardiorenal effects not established (b) Avoid in liver impairment. Self-monitor blood glucose if driving (c) Take with main meals
DPP-4 inhibitors (alogliptin, linagliptin, saxagliptin, sitagliptin, vildagliptin)	Inhibit DPP-4: prolong half-life of incretin hormones (GLP-1 and GIP), enhance prandial insulin secretion	(a) Efficacy intermediate (b) Hypo risk low (c) Weight neutral	(a) Clinically significant cardiorenal benefit not established (b) Discontinue if acute pancreatitis. Adjust dose in renal impairment except linagliptin (c) Overall good safety profile
Thiazolidinedione (pioglitazone)	Improve insulin action via PPARγ agonism	(a) Efficacy high (b) Hypo risk low (c) Weight gain	(a) Possible reduced risk of ASCVD, notably stroke (b) Slow onset of action. Risk of oedema, heart failure and bone fractures. Check liver enzymes
SGLT2 inhibitors (canagliflozin, dapagliflozin, empagliflozin, ertugliflozin)	Inhibit SGLT2 in kidney, decrease renal glucose reabsorption, increase glucose elimination in urine	(a) Efficacy intermediate to high (b) Hypo risk low (c) Weight reduction	(a) Reduce onset/progression of heart failure and CKD (b) Check for adequate renal function and hydration. Increased risk of genital and urinary infections and euglycaemic ketosis (c) Diuretic effect, reduction in blood pressure
Alpha-glucosidase inhibitors (acarbose)	Slow rate of carbohydrate digestion, reduce prandial glycaemic excursions	(a) Efficacy intermediate to low (b) Hypo risk low (c) Weight neutral	(a) Clinically significant cardiorenal benefit not established (b) Avoid if gastrointestinal disorders (c) Side effect of flatulence
Dopamine agonist (bromocriptine)	Dopamine D2 receptor agonist, reduces hepatic glucose output	(a) Efficacy intermediate (b) Hypo risk low (c) Weight neutral	(a) Clinically significant cardiorenal effects not established (b) Avoid if uncontrolled hypertension. Doses and timings used for type 2 diabetes are much lower than in treatment of Parkinson’s disease (c) Can improve circadian rhythm of glycaemic control
Bile acid sequestrant (colesevelam)	Binds bile acids in intestine, interrupts enterohepatic bile acid circulation: alters microbiome, increases secretion of GLP-1	(a) Efficacy low (b) Hypo risk low (c) Weight neutral	(a) Possible reduced risk of ASCVD (b) Avoid if obstructive gastrointestinal disease, pancreatitis, ketosis, hypertriglyceridaemia (c) Reduces hypercholesterolaemia. May interfere with absorption of vitamins and minerals
Subcutaneous injection			
GLP-1 receptor agonists (dulaglutide, exenatide QW, liraglutide, semaglutide, semaglutide oral, tirzepatide)	Activate GLP-1 receptors, potentiate glucose-induced insulin secretion, decrease glucagon secretion, increase satiety, delay gastric emptying	(a) Efficacy high or very high (b) Hypo risk low (c) Weight reduction	(a) Typically reduce albuminuria and some agents in class significantly reduce ASCVD and CV deaths (b) Initial nausea, titrate as appropriate. Discontinue if acute pancreatitis (c) Reduce blood pressure
Amylin analogue (pramlintide)	Increase satiety, delay gastric emptying, decrease glucagon secretion	(a) Efficacy intermediate (b) Hypo risk low (c) Weight reduction	(a) Clinically significant cardiorenal benefit not established (b) Initial nausea; avoid if gastroparesis, caution if hypoglycaemia unawareness (c) Only used as prandial adjunct to insulin therapy
Insulin (ultra-rapid acting: Fiasp, Lyumjev; rapid acting: Aspart, Glulisine, Lispro; short acting: Actrapid, Humulin S, Insuman Rapid; intermediate: Insulatard, Humulin I; long acting: degludec, glargine; very long acting: icodec; biphasic (premixed): Humalog, Humulin M3, Novomix)	Promote peripheral glucose uptake, glycogenesis and metabolism, decrease gluconeogenesis, increase lipogenesis, decrease lipolysis, increase protein synthesis, decrease protein catabolism, involvement in cell growth, division and differentiation	(a) Efficacy very high (b) Hypo risk high (c) Weight gain	(a) Clinically significant cardiorenal benefit not clear, although acute effects to improve vasorelaxation and reduce atherogenesis and thrombus formation (b) Important to align treatment regimen with patient lifestyle and needs. Requires appropriate lifestyle adjustments and glucose monitoring, especially if driving or operating machinery (c) High risk of hypoglycaemia: carry glucose. Increase renal sodium reabsorption

^a^Based on ADA/EASD and American Association of Clinical Endocrinology (AACE) consensus statements [2–4]

^b^Semaglutide is listed as an injection but is also available as an oral formulation. Some agents are not available in all countries; dosage forms and prescribing information listed in the summary of product characteristics may vary between countries. Additional agents (not listed here) have indications as glucose-lowering agents outside Europe and North America. Exenatide BD and lixisenatide have recently been discontinued. Tirzepatide is a GLP-1/GIP dual receptor agonist but is listed under GLP-1 receptor agonists for the purposes of this review. Fixed-dose combinations of oral agents (e.g. single tablet combinations of metformin with a DPP-4 inhibitor, sulfonylurea, pioglitazone or SGLT2 inhibitor) and fixed-ratio injectable combinations of a GLP-1RA with insulin are available. Premixed insulins and biosimilar insulins (not listed in this table) are also available. Note that in Europe the composition of a premixed insulin is described with the short-acting component before the long-acting component, whereas the description is the other way around in some countries (e.g. USA). Detemir (not listed in the table) and insulatard cartridges are being discontinued through 2025–2026

ASCVD, atherosclerotic CVD; CKD, chronic kidney disease; CV, cardiovascular; DPP-4, dipeptidyl peptidase-4; GIP, glucose-dependent insulinotropic polypeptide; GLP-1, glucagon-like peptide-1; hypo, hypoglycaemia; IR, immediate release; MR, modified release; PPARγ, peroxisome proliferator-activated receptor-gamma; QW, once weekly; SGLT, sodium–glucose cotransporter; SR, sustained release; XR, extended release

This narrative review considers potential new non-insulin pharmacological approaches to glycaemic control including agents in clinical development and examples of preclinical research that illustrate novel mechanisms. Lessons learned from several initiatives that have not proceeded in development are described in ESM Appendix 3.

## Unmet needs

In principle, an understanding of disease pathophysiology and the limitations of existing therapies (ESM Appendix 2) will identify unmet needs and point to potential novel pharmacological targets. However, the history of diabetes medications illustrates how clinical serendipity has been as productive as molecular modelling, logical scientific design and mass screening for active compounds [5]. Current clinical guidance reminds us that phenotypic presentations of type 2 diabetes are highly variable but that there are common unmet needs and conformities in therapeutic strategy [2–4]. Hyperglycaemia (as a marker of defective glucose homeostasis) is a fundamental feature linked to retinopathic, neuropathic and nephropathic complications, and a contributing risk for associated morbidities, particularly cardiovascular diseases. Thus, improved glycaemic control is recognised as a crucial therapeutic objective. Accordingly, the areas of unmet need for new treatments are reflected in the lesions that underly the hyperglycaemia, notably impaired function and number of pancreatic beta cells, insulin resistance and glucose toxicity, which are frequently aggravated by excess adiposity, abnormalities of glucagon secretion, an altered incretin response and disturbances in lipid metabolism [6].

Regarding pancreatic beta cell defects, there are particular needs to restore first-phase glucose-induced insulin release and improve proinsulin-to-insulin processing, as neither problem is adequately addressed by existing therapies. General interventions are required to protect against cytotoxic damage to beta cells (e.g. by proinflammatory factors and products of metabolic stress) and specifically to counter the declining beta cell population using therapies that reduce beta cell apoptosis or replenish through beta cell neogenesis.

Insulin resistance impairs nutrient metabolism and vascular function but usually responds well to reduced adiposity. However, additional therapies are required to circumvent rate-limiting steps within the signalling pathways of insulin action or to intervene directly in cellular glucose metabolism to reduce excess glucose production, improve peripheral glucose disposal and reduce glucotoxicity. Excess adiposity is a particular overarching challenge in the treatment of type 2 diabetes because weight control can benefit so many of the other unmet needs by reducing metabolic stress to beta cells, improving insulin sensitivity and independently reducing the risk of associated morbidities [6]. Recently available satiety-inducing incretin-based medicines are already revolutionising obesity management and greatly improving glycaemic control in type 2 diabetes [7].

**Fig. 1:**
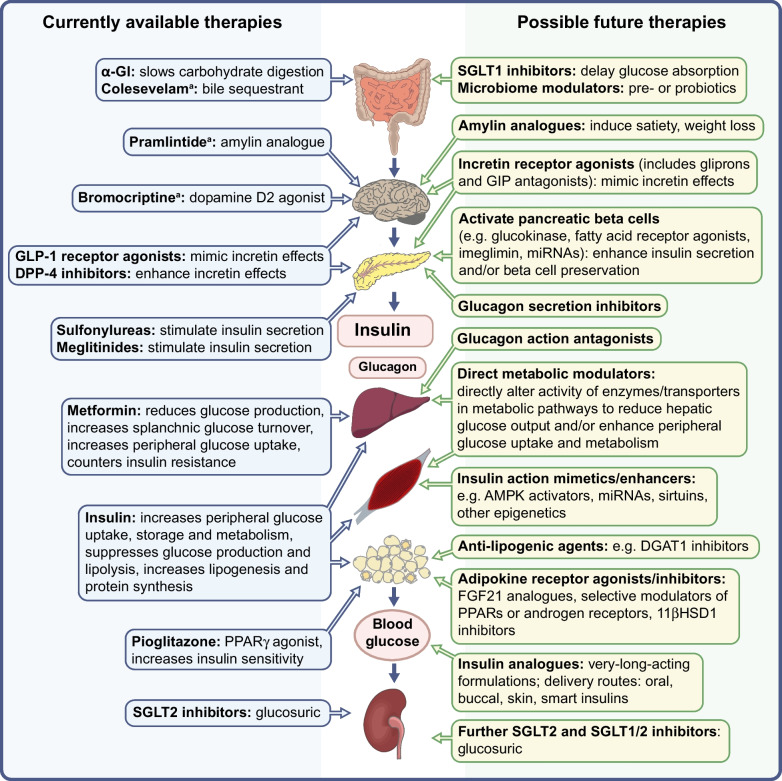
Sites of action of currently available and potential future glucose-lowering agents for use in the management of type 2 diabetes. ^a^Not indicated for glucose-lowering in Europe. 11βHSD1, 11β-hydroxysteroid dehydrogenase 1; α-GI, alpha-glucosidase inhibitor; AMPK, adenosine monophosphate-activated protein kinase; DGAT1, diacylglycerol acyltransferase 1; DPP-4, dipeptidyl peptidase-4; FGF21, fibroblast growth factor 21; GIP, glucose-dependent insulinotropic polypeptide; GLP-1, glucagon-like peptide-1; PPAR, peroxisome proliferator-activated receptor; QW, once weekly; SGLT, sodium–glucose cotransporter. Adapted from Bailey et al [99] with permission from Elsevier. This figure is available as a downloadable slide

An ideal new agent will provide durable blood glucose-lowering efficacy that is better than or similar to that of available agents, carry minimal risk of overt hypoglycaemia, facilitate weight control and hopefully provide some further advantages, particularly against associated morbidities. Such an agent will probably have a different and complementary mode of action that enables use in combination therapy, and/or be conducive to use in clinical circumstances that are less well served by existing agents, for example in obesity, atherosclerotic CVD, heart failure, chronic kidney disease, frail sarcopenia and other conditions commonly encountered among those with type 2 diabetes [1]. Tolerability, ease of administration, minimal monitoring requirements and cost are also important determinants of acceptability, and preventive advantage for individuals with impaired glucose tolerance would be very desirable. Above all, the long-term safety profile must provide a strongly favourable balance of benefit over risk. A summary of potential agents considered in the following sections is provided in Fig. 1.

While new therapies can address aspects of need relating to disease pathophysiology, they do not necessarily obviate barriers that are known to preclude optimal use of existing therapies [8]. For example, from the patient perspective, inadequate health literacy, financial constraints, phobias, fear of hypoglycaemia, and social, cultural or personal pressures can adversely impact lifestyle and treatment adherence. From a clinician perspective, inappropriately structured healthcare provision, scarcity of resources, educational requirements, delayed diagnosis and treatment inertia also represent significant challenges. Advances in drug delivery (e.g. slow-release tablet formulations, once-weekly injections, pumps and transdermal patches), fixed-dose combinations, the reassurance of continuous glucose monitoring, digital aids and educational programmes can reduce some of these barriers, but new therapies alone cannot guarantee universal appeal, acceptance or proper use.

## Pragmatic considerations

Although the underlying principles of drug development are conveniently categorised into a series of preclinical and clinical phases with recognised achievement criteria, a new medicine for type 2 diabetes must accommodate several particularly complex challenges [9]. The progressive heterogeneous nature of type 2 diabetes and its broad pathogenic impact may commit a new medicine to lifelong use through varying clinical circumstances that impose diverse safety constraints. Thus, regulatory approval is subject to predefined cardiovascular safety analyses and usually a post-approval dedicated cardiovascular outcomes trial as well as studies in potentially vulnerable subgroups such as those with chronic kidney disease [3, 4, 9]. For examples of requirements for regulatory approval, see [10, 11]. Development of a new agent from discovery to market usually takes about 10–12 years, but this timespan can vary significantly. Estimating the cost of research and development for a new agent is complicated by the compounds included and discarded in the process. Phase 3 trials account for the majority of pre-approval expenditure, and estimates of US$ 1–2.7 billion have been suggested to account for overall expenditure up to completion of submission for regulatory approval [12].

## Insulin secretion and beta cell preservation

Many in vitro and preclinical studies have examined agents that enhance insulin secretion, including agents that generate ATP, inhibit K^+^-ATP channels, raise cytosolic calcium, activate muscarinic or imidazoline receptors, suppress α2-adrenergic receptors, inhibit phosphodiesterases to increase cAMP, stimulate phospholipases or modulate various intracellular regulators of insulin exocytosis. However, it has not been possible to specifically target these agents in pancreatic beta cells and exclude extraneous effects [1, 9]. Another problem is that preclinical research has mostly studied interventions at early stages of diabetic syndromes that do not readily apply to more advanced stages of clinical disease. In addition, differences between autoimmune beta cell loss in type 1 diabetes and metabolically driven beta cell loss in type 2 diabetes have warranted different therapeutic approaches to beta cell preservation, although some future initiatives for beta cell neogenesis may be suitable for the treatment of both types of diabetes [13].

### Imeglimin

Imeglimin is a tetrahydrotriazine that was recently approved to treat type 2 diabetes in Japan (2021) and India (2022) and which has been shown to improve glucose-induced insulin secretion, especially first phase, in clinical trials [14]. It increases the production of nicotinamide adenine dinucleotide (NAD) via nicotinamide phosphoribosyltransferase (NAMPT) and increases the activity of the mitochondrial respiratory chain to promote ATP synthesis and reduce oxidative stress [15]. Preliminary studies in other tissues suggest that high concentrations of imeglimin may increase the expression of genes encoding respiratory chain complexes 1 and 3 [16]. Imeglimin reduces beta cell apoptosis and maintains beta cell mass when introduced early in the development of diabetes in animal models, possibly by reducing apoptotic cues via effects on mitochondrial viability, but it is not known whether such protection operates in advanced clinical disease [17]. Imeglimin has also been reported to reduce lipotoxicity and inflammation and improve insulin sensitivity [18].

### Glucokinase activators

Because the control of glucokinase differs between pancreatic beta cells and liver, allosteric activators (selected to increase glucose affinity) can enhance insulin secretion (including first phase) without an excessive increase in glucose uptake and use by the liver [19, 20]. With careful dose titration and timing to align with meals, the risk of insulin oversupply and hypoglycaemia can be minimised, and several glucokinase activators with variable effects on beta cells vs liver have improved glycaemic control in clinical studies [21]. Dorzagliatin activates glucokinase in pancreas and liver and is approved in China (2022). It enhances the secretion of insulin and glucagon-like peptide-1 (GLP-1) and reduces glucagon secretion. In a 24 week Phase 3 randomised double-blind placebo-controlled trial in drug-naive individuals with type 2 diabetes, dorzagliatin lowered HbA_1c_ by ~0.6 mmol/mol (0.57%; placebo subtracted) and did not increase hypoglycaemia [22]. In preclinical studies dorzagliatin improved beta cell mass, but most long-term (>6 months) clinical studies with glucokinase activators have noted a loss of effectiveness, providing a reminder that persistently increased glucose stimulation of the beta cell may hasten functional exhaustion and demise [20, 23].

### Fatty acid receptor agonists

Pancreatic beta cells and intestinal L cells express several types of G protein-coupled receptors for fatty acids that potentiate the secretion of insulin and GLP-1 [24]. Selective small molecule agonists have been developed to activate many of these receptors, notably G protein-coupled receptor 40 (GPR40 [free fatty acid receptor (FFAR) 1]), GPR119 and GPR120 (FFAR4), and several have shown efficacy in animal models. However, efficacy in clinical studies has been disappointing, possibly because excessive chronic stimulation is associated with receptor internalisation, and some agents have disturbed liver function [25, 26].

### miRNAs

The expression of many miRNAs in pancreatic beta cells is altered in diabetic states, providing a potential (if still highly challenging) therapeutic approach [27]. An example of overexpression of an miRNA in type 2 diabetes is miR-200c, which decreases the expression of ETS variant transcription factor 5 (ETV5), causing disruption to insulin exocytosis. Inhibition of miR-200c using an antagomir with complementary nucleotides increased glucose-induced insulin secretion (almost threefold) in islets from type 2 diabetes donors [28]. Several miRNAs are known to enhance insulin production, reduce beta cell loss and/or promote beta cell proliferation, such as miR-30d (via increased insulin gene expression) and miR-375 (mechanism unclear) [27]. However, delivering miRNAs or inhibitors specifically to beta cells remains a major hurdle for the clinical application of this therapeutic approach [29]. Similarly, the therapeutic potential of miRNA targets to improve insulin action has been compromised by difficulty in restricting agents to selected tissues (ESM Appendix 3).

### Other activators of pancreatic beta cells

Incretin hormone receptor agonists (discussed below) can reduce beta cell apoptosis in vitro and in animal models, probably via cAMP and protein kinase A (PKA) activation, and provide the best evidence-supported opportunity to preserve the beta cell population at this time. Indeed, GLP-1 receptor agonists (GLP-1RAs) sustain insulin secretion during long-term clinical use, consistent with a direct benefit to the beta cell population [7]. An extensive list of agents including thiazolidinediones, insulin-like growth factors, fibroblast growth factors, gastrin, glycogen synthase kinase-3β (GSK-3β) inhibitors, protein kinase dual-specificity tyrosine-phosphorylation-regulated kinase 1A (DYRK1A) inhibitors, gamma-aminobutyric acid, quinolones, sphingosine phosphate, oestrogens and phytoestrogens, polyphenols, flavonoids and other plant compounds has been reported to increase beta cell proliferation in vitro or in animal models, but specifically targeting these agents to pancreatic beta cells in vivo remains an unfulfilled therapeutic challenge [30].

### Cell-based therapies

Although cell-based therapies exceed the remit of this review, some cell-based technologies that might be delivered non-surgically and applied to type 2 (and type 1) diabetes are briefly mentioned here [13]. Preventing beta cell de-differentiation, stimulating proliferation and differentiation of pluripotent stem cells, and enhancing CRISPR-targeted gene editing can be mediated with small molecules and used in conjunction with conventional glucose-lowering therapies. It has long been known that pancreatic centroacinar cells can give rise to new insulin-secreting cells, and preclinical studies have noted that bone morphogenetic protein-7 (BMP7) can induce beta cell neogenesis from pancreatic ductal tissue. Agents that mimic BMP7 or promote BMP7 signalling via the BMP receptor activin-like kinase 3 (ALK3) are being assessed as another (potentially less expensive) approach to islet regeneration [31]. For further information, see [32–34].

## Glucagon antagonists

Interventions that suppress prandial glucagon secretion can reduce hyperglycaemia in type 2 diabetes, but timed targeting specifically to pancreatic alpha cells in vivo has proved difficult. Antagonising glucagon receptors (GCGRs) with modified glucagon analogues, small molecules or antisense oligonucleotides has also been problematic because of the risk of hypoglycaemia and adverse effects on liver function, including steatosis and an increase in LDL-cholesterol. There is also a compensatory increase in glucagon secretion, which can cause an acute rebound hyperglycaemia if treatment is missed or stopped [35, 36].

## GLP-1 receptor agonists

GLP-1 became the focus of incretin therapy because of its strong potentiation of glucose-induced insulin secretion and glucose-lowering efficacy in type 2 diabetes. Accordingly, several analogues (once daily and once weekly subcutaneously injected and once daily oral) with potent GLP-1RA effects are well established within treatment algorithms [2–4, 7]. Use of a GLP-1RA can assist remission from early-stage disease and provide sustained benefits throughout the natural history of the disease, including compatible use with other classes of glucose-lowering agents. Weight loss in particular, but also cardiorenal benefits and other potential advantages, have encouraged wider use, and several further GLP-1RAs with ever longer circulating half-lives are advancing in development, aiming to leverage the convenience of administration with minimal gastrointestinal side effects (Table 2) [37].

**Table 2:** Incretin-based and amylin-based peptide and non-peptide (small) molecules in development for the management of hyperglycaemia in individuals with overweight/obesity and type 2 diabetes

Agent (sponsor)	Receptor targets	Route	Timing	Phase^a^	Participants, trial duration, primary trial results^b,c^	Reference
Peptide						
Amycretin (Novo)	AmylinRA/GLP-1RA	Oral	OD	1	OW/O, 12 weeks ↓BW 13.1%	[69]
Cagrilintide + semaglutide (CagriSema) (Novo)	AmylinRA + GLP-1RA mix	SC	QW	2	OW/O T2D, 32 weeks ↓HbA_1c_ 2.2%^d^, ↓BW 15.6%^b^	[67]
		SC	QW	3	OW/O, 68 weeks ↓BW 20.4%	[68]
		SC	QW	3	OW/O T2D, 68 weeks ↓HbA_1c_ 1.4%, ↓BW 10.4%	[68]
CT-388 (Roche)	GLP-1RA/GIPRA	SC	QW	1	OW/O, 24 weeks ↓BW 18.8%	[91]
Ecnoglutide (Sciwind)	GLP-1RA	SC	QW	2	NW/OW T2D, 20 weeks ↓HbA_1c_ 2.39%, ↓BW 2.26 kg	[41]
Maridebart cafraglutide (MariTide, AMG133) (Amgen)	GLP-1RA/GIPRi	SC	QM	2	OW/O T2D, 52 weeks ↓HbA_1c_ ~1.5%, ↓BW 10.6%	[55]
		SC	QM	2	OW/O, 52 weeks ↓BW 13.7%	[55]
Mazdutide (Lilly)	GLP-1RA/GCGRA	SC	QW	3	OW/O, 48 weeks ↓BW 13.3%	[59]
Pemvidutide (Altimmune)	GLP-1RA/GCGRA	SC	QW	2	OW/O MASLD ± T2D, 12 weeks ↓BW 3.5%, rrLFC 57.1%	[58]
Retatrutide (Lilly)	GLP-1RA/GIPRA/GCGRA	SC	QW	2	OW/O T2D 24 weeks ↓HbA_1c_ 2.01% 36 weeks ↓BW 13.9%	[61]
		SC	QW	2	OW/O, 48 weeks ↓BW 22.1%	[92]
		SC	QW	2	OW/O MASLD, 24 weeks ↓BW 17.6%, rrLFC 82.4%	[93]
Survodutide (BI)	GLP-1RA/GCGRA	SC	QW	2	OW/O, 46 weeks ↓BW 14.9%	[57]
VK2735 (Viking)	GLP-1RA/GIPRA	SC	QW	2	OW/O, 13 weeks ↓BW 13.1%	[94]
		Oral	OD	2	OW/O, 13 weeks ↓BW 10.9%	[95]
ZT002 (Beijing QL)	GLP-1RA	SC	QM	1	OW/O, 14 weeks ↓BW 13.1%	[40]
Non-peptide						
Orforglipron (Lilly)	GLP-1RA	Oral	OD	2	NW/OW/O T2D, 26 weeks ↓HbA_1c_ 1.67%, ↓BW 7.9 kg	[96]
		Oral	OD	3	OW/O T2D, 40 weeks ↓HbA_1c_ 1.07%, ↓BW 5.9 kg	[83]
Aleniglipron (Structure)	GLP-1RA	Oral	OD	2	OW/O, 12 weeks ↓BW 6.2%	[97]
CT-996 (Roche)	GLP-1RA	Oral	OD	1	OW/O, 4 weeks ↓BW 6.1%	[85]
CX11/VCT220 (Corxel)	GLP-1RA	Oral	OD	2	OW/O, 16 weeks ↓BW 8.1%	[86]
Tern-601 (Tern)	GLP-1RA	Oral	OD	1	OW/O, 4 weeks ↓BW 5.0%	[98]

^a^For Phase 3 RCTs, efficacy was calculated as placebo-subtracted change in HbA_1c_ or body weight for the top dose tested

^b^In trials involving individuals with type 2 diabetes, the test agent or placebo was administered as an add-on to lifestyle (diet + exercise) or lifestyle plus metformin. In trials involving individuals without diabetes, the test agent or placebo was administered as an add-on to lifestyle

^c^HbA_1c_ decreases are reported in per cent as in most of the original reports. To convert HbA_1c_ values from % to mmol/mol in this table, multiply by 10.929. Thus, 1% is equivalent to 10.929 mmol/mol. This is different to the conversion for absolute values in blood that exceed ~3%, which require multiplication by 10.929 and subtraction of 23.5

^d^Not placebo subtracted as there was not a placebo arm in this trial, which compared cagrilintide + semaglutide against cagrilintide or semaglutide alone

BI, Boehringer Ingelheim; BW, body weight; MASLD, metabolic dysfunction-associated steatotic liver disease; mix, mixture within same injection, NW, normal weight; O, obesity; OD, once daily; OW, overweight; QM, once monthly; QW, once weekly; SC, s.c. injection; RA, receptor agonist; Ri, receptor inhibitor; rrLFC, relative reduction in liver fat content; T2D, type 2 diabetes; ↓, decrease

Adapted from Bailey et al [37] under the terms of the Creative Commons CC-BY licence (https://creativecommons.org/licenses/by/4.0/)

Efpeglenatide (a modified exendin conjugated to human immunoglobulin-4 via a polyethylene glycol linker) has shown metabolic efficacy and cardiorenal benefits as a once-weekly subcutaneous injection during Phase 3 trials, but has yet to be progressed further [38]. However, efpeglenatide was also effective as a biweekly and once-monthly subcutaneous injection, illustrating the opportunity to produce ever longer-acting GLP-1RAs for less frequent administration [39]. Another very-long-acting GLP-1RA is ZT002, a GLP-1 analogue with a C-terminal extension to increase stability and two C-18 fatty acid chains to increase attachment to albumin. It has a circulating half-life of ~12 days, which enables biweekly or once-monthly subcutaneous injection [40]. For increased potency, ecnoglutide is a GLP-1 analogue with an Ala8 to Val substitution and a C-18 fatty acid at Lys30. This enables biased agonism at the GLP-1 receptor through increased signalling via cAMP and reduced recruitment of β-arrestin, thereby reducing receptor internalisation [41].

The concept of continuous slow-release delivery of a GLP-1RA has been assessed in a trial of the Intarcia ITCA 650. This subcutaneously implanted titanium osmotic mini-pump released exenatide and reduced HbA_1c_ by ~13 mmol/mol (1.2%) over 39 weeks compared with placebo in participants with type 2 diabetes; however, the device has not been approved by the US Food and Drug Administration (FDA) [42]. Various biodegradable and low- or non-immunogenic subcutaneous depots have undergone preclinical testing for slow release of GLP-1 and GLP-1 analogues, including microspheres and nanoparticles of poly-lactide-glycolide (PLGA) and modified hydrogel reservoirs [43]. Linkage of GLP-1 into elastin-like protein (ELP) polymers that form viscous reservoirs at body temperature and methods for enzyme-controlled release of GLP-1 from reservoirs have also been described but have yet to receive clinical evaluation [44]. Regarding non-invasive delivery of peptide GLP-1RAs, formulation for oral administration using the absorption enhancer sodium caprylate (SNAC) to facilitate uptake of semaglutide by the stomach is well established, and inhaled and transdermal patch delivery systems are also being tested [45, 46].

Looking beyond GLP-1 receptor mono-agonists, the design of future incretin-based peptide therapies is destined to provide multifunctional capability, such that a single peptide can interact with several different receptors to create a broad portfolio of effects [37].

### GLP-1 receptor agonist cautions

Because use of GLP-1RAs is hampered by gastrointestinal and biliary side effects, various dose escalation strategies and dietary adaptations have been implemented. However, future molecules may be able to alleviate this issue via modified effects on neural pathways controlling gastric emptying. There is a risk of hypoglycaemia with over-aggressive use of combination therapies that include a GLP-1RA, even though the individual agents do not carry this risk. In addition, reduced appetite with use of GLP-1RAs requires attention to adequate intake of vitamins, minerals, protein, fibre and fluids. Rapid lowering of hyperglycaemia has been associated with progression of retinopathy in some studies, and long-term use of incretin therapy may be linked to rare cases of non-arteritic anterior ischaemic optic neuropathy (NAION). Debate regarding management of therapy when targets for weight and blood glucose have been achieved currently favours continuation with a low-dose regimen for most individuals [37].

Loss of muscle mass, especially with rapid weight reduction during GLP-1RA therapy, has prompted studies into the utility of agents that promote muscle growth. Initial studies have indicated preservation of muscle mass when semaglutide therapy is combined with an anti-growth differentiation factor 8 (GDF8)/anti-myostatin antibody (trevogrumab) or an anti-activin A antibody (garetosmab) [47]. Other activin receptor inhibitors such as the monoclonal antibody bimagrumab are also being considered to preserve muscle mass during use of weight loss treatments [48].

## GIP/GLP-1 receptor co-agonists

The incretin hormone glucose-dependent insulinotropic polypeptide (GIP) was not initially adopted as a therapeutic because it loses glucose-lowering efficacy in type 2 diabetes [7, 49]. Potentiation of glucose-induced insulin secretion by GIP is diminished in chronic hyperglycaemic states due at least in part to a decrease in the Gs subtype of the GIP receptor in pancreatic beta cells, and further reasons for concern over GIP as a treatment for type 2 diabetes are that it increases glucagon secretion and adipose deposition [49]. However, delivery of a GIP receptor agonist with a GLP-1RA in approximately physiological proportions results in an additive glucose-lowering and weight-lowering effect [49].

### Interactions of GIP with GLP-1

Relevant to GIP–GLP-1 interaction is evidence that GIP receptor gene polymorphisms can alter receptor signalling sufficiently to modify the risk and progression of type 2 diabetes [50]. It is also possible that chronic GIP receptor agonism promotes receptor desensitisation or a biased agonism effect that gives rise to functional suppression of GIP activity and enhanced GLP-1 activity [49, 51]. Indeed, preclinical studies have shown that both agonism and antagonism of GIP receptors can reduce hyperglycaemia and body weight [49, 52]. The agonism–antagonism conundrum with GIP is further complicated by species-specific differences in GIP receptor interactions, which have interfered with the usual extrapolation of preclinical findings into clinical drug development [37, 49, 52].

Despite the GIP conundrum, the first approved GIP receptor/GLP-1 receptor co-agonist tirzepatide has confirmed the efficacy gains of the dual agonism effect: placebo-subtracted decreases in HbA_1c_ >22 mmol/mol (>2%) and body weight >8 kg were achieved during 40 week clinical trials in participants with overweight/obesity and type 2 diabetes [53]. Accordingly, several further synthetic peptides that are GIP receptor/GLP-1 receptor co-agonists are advancing in preclinical and early clinical development; for example, LY3537031 is designed for subcutaneous injection while VK2735 and NN0519-0130 include both injectable and oral formulations (Table 2) [37].

## GIP receptor antagonists

Given the reductions in blood glucose and body weight afforded by GIP receptor antagonists in experimental studies, a bispecific monoclonal anti-human GIP receptor antagonist antibody covalently linked to two GLP-1RAs (maridebart cafraglutide, known as MariTide or AMG133) has been developed as a once-monthly subcutaneous injection [54]. In a Phase 1 trial, three once-monthly injections of 420 mg of maridebart cafraglutide in overweight/obese individuals without diabetes reduced body weight by 14%, and most of the weight loss was maintained for 2–3 months after treatment was discontinued [54]. In a 52 week Phase 2 trial, overweight/obese individuals without diabetes lost 13.7% of their body weight (placebo subtracted) after receiving maridebart cafraglutide (420 mg once monthly), and overweight/obese individuals with diabetes lost 10.6% of their body weight and saw a decrease in HbA_1c_ of ~17 mmol/mol (1.5%, placebo subtracted) after receiving maridebart cafraglutide at the same dose (Table 2) [55]. Other approaches to suppress GIP action (yet to undergo clinical trials) include various GIP receptor antagonist peptides, a small molecule GIP receptor antagonist (SKL-14959) and an anti-GIP monoclonal antibody (HCR-188) [51, 56].

## Glucagon receptor agonists

Co-agonist and multi-agonist synthetic incretin-based peptides incorporating GCGR agonism are now advancing in pharmaceutical pipelines. These include the GLP-1 receptor/GCGR co-agonists survodutide [57], pemvidutide [58] and mazdutide [59] as well as GIP receptor/GLP-1 receptor/GCGR triple agonists (e.g. retatrutide and efocipegtrutide) (Table 2) [37]. In view of the blood glucose-raising effect of glucagon, the value of GCGR agonism in these therapies might be questioned. However, several of these agents have already shown substantial glucose-lowering efficacy, and it is likely that increased energy expenditure and weight reduction are conferred by GCGR agonism, while GLP-1 receptor agonism suppresses endogenous glucagon secretion [37]. Some of these agents may provide particular benefits in the treatment of metabolic dysfunction-associated steatotic liver disease (MASLD), as they produce substantial reductions in liver fat content, and others may be more suited to the treatment of obesity without diabetes [60]. However, the GLP-1 receptor/GIP receptor/GCGR triple agonist retatrutide showed strong glucose-lowering and weight-lowering efficacy (HbA_1c_ reduction of 22.4 mmol/mol [2.01%] at 24 weeks and body weight reduction of 13.9% at 36 weeks, placebo subtracted) during a Phase 2 trial in overweight/obese individuals with type 2 diabetes [61]. The GLP-1 receptor/GIP receptor/GCGR triple agonist efocipegtrutide is presently under investigation in individuals with MASLD, and preclinical accounts of other dual and triple agonists that include GLP-1 receptor and GCGR agonism suggest that clinical studies in individuals with type 2 diabetes will follow [62, 63].

## Amylin receptor agonists

The use of non-aggregating amylin analogues to reduce body weight and blood glucose through increased satiety, delayed gastric emptying and suppression of glucagon secretion is illustrated by pramlintide, which has been available in some regions since 2005 as an injectable adjunct to insulin therapy [64]. These effects are centrally mediated via the amylin receptor AMY1 (and to a lesser extent the AMY2 and AMY3 receptors) outside the blood–brain barrier in the area postrema. The current focus of research is on the treatment of obesity with long-acting non-precipitating amylin analogues that provide varying selectivity for the different AMY receptors. Examples of agents receiving initial clinical assessment are eloralintide, petrelintide, amylin 355 and 1213, AZ06234, ZP8396 and MET2331. The interaction of calcitonin with a separate part of the AMY receptors does not appear to be affected by interaction with the amylin analogues [64–66].

A long-acting amylin analogue, cagrilintide, which has an N-terminal C-20 fatty acid chain for albumin binding, has shown strong weight-lowering properties, and once-weekly subcutaneous injection of cagrilintide with semaglutide (CagriSema; each at a dose of 2.4 mg) reduced HbA_1c_ by ~24 mmol/mol (2.2%) and body weight by 15.6% (vs baseline) during a 32 week Phase 2 trial in overweight/obese adults with type 2 diabetes [67]. In two Phase 3 studies, a fixed-dose combination of cagrilintide and semaglutide (each at 2.4 mg) was associated with weight reductions of 20.4% and 10.4% (placebo subtracted) after once-weekly subcutaneous injection for 68 weeks in overweight/obese individuals without and with type 2 diabetes, respectively (Table 2) [68].

The activation of AMY and GLP-1 receptors has been further evaluated in preclinical studies using single peptide AMY receptor/GLP-1 receptor co-agonists and in a clinical study with the co-agonist amycretin [64]. When amycretin (50 mg) was delivered as an oral formulation using the gastric absorption enhancer SNAC during a 12 week Phase 1 trial in individuals with obesity, body weight was reduced by 13.1%, boding well for longer-term efficacy [69].

Because AMY receptors also interact with calcitonin, dual amylin/calcitonin receptor agonists (DACRAs) have been developed, with preclinical studies finding greater weight loss than with amylin analogues alone, and additional efficacy in combination with a GLP-1RA [70]. Multi-agonist peptides that incorporate AMY receptor agonism have also been developed as illustrated by PTT-A, a long-acting tetra-agonist at the GLP-1, GIP, amylin and calcitonin receptors that decreased food intake and body weight in a rat model of obesity [71].

## Further peptides under investigation

In addition to the receptor agonists described above, several other peptide analogues are receiving preclinical evaluation as potential glucose-lowering and weight-lowering therapies. Examples include peptide tyrosine tyrosine (PYY), pancreatic polypeptide, cholecystokinin (CCK), GLP-2 and secretin, which suppress food intake, mostly via centrally mediated mechanisms [72–76]. Ghrelin receptor antagonists, modulators of neuropeptide Y receptors and activators of neurokinin-2 receptors have also received attention as appetite suppressants and promoters of energy expenditure [74, 74, 77]. The potential importance of assessing peptides from the intestinal tract, in particular, is illustrated by the fact that surgical procedures that bypass, damage or prevent nutrient contact with the duodenal mucosa are all known to improve blood glucose and body weight control in type 2 diabetes [37].

Administration of fibroblast growth factor-21 (FGF21) and analogues and antibodies that exhibit agonism of the fibroblast growth factor receptor 1 (FGFR1)–Klotho beta (KLB) receptor complex can reduce body weight, liver fat, insulin resistance and blood glucose in animal models of obesity and diabetes, and initial clinical studies have encouraged further interest [77]. Fusion proteins linking GLP-1 and FGF21 analogues have shown efficacy in treating MASLD. Although previous trials of leptin receptor (LepR) agonists have failed because of leptin resistance, a LepR/GLP-1 receptor co-agonist has undergone preclinical study [78]. Preclinical assessments of other novel co-agonists that incorporate GLP-1 receptor agonism, include a GLP-1 receptor/CCK receptor co-agonist and a GLP-1 receptor/gastrin receptor co-agonist [79, 80].

## Gliprons

Small molecule GLP-1RAs (‘gliprons’) have been studied in vitro and after oral dosing in animal models, but few have progressed in clinical development due to limitations of potency and possible adverse liver effects (Table 2) [81, 82]. Of note, however, in a 40 week Phase 3 study of overweight/obese individuals with type 2 diabetes, 36 mg of orforglipron taken orally once daily achieved reductions in HbA_1c_ of ~12 mmol/mol (1.07%) and in body weight of 5.9 kg (placebo subtracted) with an adverse event profile similar to that of the established GLP-1RA class [83]. Several oral small molecule GLP-1RAs are presently in early clinical development, including CX11 (VCT220), HRS-7535 and CT-996; the last has shown biased agonism with reduced β-arrestin-mediated receptor internalisation in preclinical studies [84–86]. Small molecule positive allosteric modulators of the GLP-1 receptor, with or without their own agonist capability, can alter the conformation of the GLP-1 receptor and increase its affinity for GLP-1. Whether allosteric receptor modulators could be exploited for therapeutic gain awaits further study [81, 87].

## Insulin action enhancers

Although insulin resistance is an underlying feature of most presentations of type 2 diabetes, it may involve multiple defects, which complicates therapeutic targeting, and may extend beyond nutrient metabolism to include vascular activities, growth, electrolyte control and other physiological functions. Potential therapeutic approaches have included activating or prolonging activation of the insulin receptor, promoting signalling through post-receptor pathways and increasing the activity of biological effectors. However, rate-limiting signalling ‘bottlenecks’ and negative feedback associated with more distal steps in the pathways have diminished the ability of otherwise promising interventions to mimic or enhance early steps in the insulin signalling pathways. In consequence, clinical exploitation of attractive research has been frustrated as described in ESM Appendix 3.

## SGLT inhibitors

Sodium–glucose cotransporter (SGLT) 2 inhibitors reduce glucotoxicity by inhibiting renal glucose reabsorption and also provide cardiorenal protective effects. Four SGLT2 inhibitors are currently available in Europe and North America; several others are available in other regions and others are in development. Some of these agents can also slow intestinal glucose absorption through a modest inhibitory effect on SGLT1, and balanced dual SGLT1/2 inhibitors such as sotagliflozin have been developed that have metabolic and cardiorenal benefits [88]. The cardiorenal protection conferred through SGLT2 and SGLT1/2 inhibition favours use in combination with other classes of glucose-lowering agents.

Preclinical studies have raised interest in developing inhibitors of other types of SGLTs. Attention has focused on inhibition of SGLT4 (in intestine and kidney) and particularly SGLT5 (in kidney) to reduce intake and increase elimination of fructose [89]. It is anticipated that this will improve insulin sensitivity and reduce fatty liver disease, hyperglycaemia and salt-sensitive hypertension.

## Other putative glucose-lowering therapies

Insights from genomics, particularly the many genetic variants associated with type 2 diabetes, and from metabolomics and bioinformatics have indicated further potential therapeutic targets, but as yet these have not led to specific pharmacological interventions [90]. Several adipokines with effects on insulin action, energy metabolism or appetite control have been studied, but none has succeeded beyond initial clinical trials (ESM Appendix 3). Lessons learned from other preliminary or unsuccessful interventions are also considered in ESM Appendix 3, including modulators of miRNAs, selective peroxisome proliferator-activated receptor modulators, hydroxysteroid dehydrogenase-1 inhibitors, adenosine monophosphate-activated protein kinase activators, direct modulators of glucose metabolism, regulators of diacylglycerol acyltransferase, sirtuins, selective androgen receptor modulators and agents to modify the microbiome.

## Conclusion

Glycaemic control is a fundamental pillar in the management of type 2 diabetes; however, although several classes of glucose-lowering agents are available to address different aspects of disease pathophysiology, many individuals with type 2 diabetes do not achieve or maintain adequate control. The virtues of weight management are also well recognised, and multi-agonist incretin-based and amylin-based synthetic peptides are addressing unmet needs, particularly regarding weight control and beta cell function. Using injections (weekly/monthly) or tablet formulations (daily), these peptides enable simultaneous engagement of several targets, conferring benefits against metabolic defects plus cardiorenal diseases, fatty liver, cravings and other complications commonly associated with type 2 diabetes. However, achievements of incretin-based therapies are often tempered by the need to maintain lifestyle changes and by gastrointestinal disturbances that compromise dose escalation, tolerability and adherence, indicating challenges for the design of future agents. In addition, although large-scale peptide production is facilitated by solid-state synthesis procedures, high costs are inevitably reflected in high prices for patients. Small molecules that substitute for peptides at receptor targets are emerging in pharmaceutical pipelines, assisted in design by advanced imaging technologies. Small molecules raise the prospect of less expensive medicines, but (unlike multi-agonist peptides) require a different molecule to interact with each type of receptor, suggesting that fixed-dose combination tablets may become more fashionable to service multi-drug strategies. Of note for drug development programmes is that guidelines and regulatory agencies are placing ever stronger emphasis on the delivery of benefits beyond glycaemic control, particularly weight control and cardiorenal protection, and on predicting best responders as part of the quest for precision medicine.

Many promising preclinical approaches to preserve beta cells, relieve insulin signalling ‘pinch points’ or directly modify intermediary metabolism have been stalled by unwanted off-target effects and have not proceeded through clinical development. New technologies including miRNAs, epigenetic modulators and artificial intelligence-guided compound design are in their infancy and have yet to supply agents to fill treatment gaps, and agents to drive beta cell neogenesis are a distant (but not unrealistic) prospect. Future attention to disease prevention could accommodate approaches beyond conventional glucose and weight control that consider how inflammatory, infective and environmental stress factors contribute to type 2 diabetes. In addition, with early diagnosis comes the opportunity for prompt use of agents to assist at least with temporary remission and defer complications; however, a permanent cure is not in sight. In the meantime, it is timely to reinforce the message in guidelines to use currently available medicines as effectively as possible to counter the ever-present challenge of ‘hyperglycaemia + time = complications’.

## Supplementary Information

Below is the link to the electronic supplementary material.

ESM (PDF 275 KB)

Figure slide (PPTX 297 KB)

## Data Availability

This article uses only data extracted from sources in the published public domain.

## Abbreviations

CCK: Cholecystokinin
FFAR: Free fatty acid receptor
FGF21: Fibroblast growth factor-21
GCGR: Glucagon receptor
GIP: Glucose-dependent insulinotropic polypeptide
GLP-1: Glucagon-like peptide-1
MASLD: Metabolic dysfunction-associated steatotic liver disease
SGLT: Sodium–glucose cotransporter
SNAC: Sodium N-(8-[2-hydroxybenzoyl] amino) caprylate

## Author’s relationships and activities

CJB has undertaken consultancy for various pharmaceutical companies involved in the development of medicines for the treatment of diabetes. CJB has also served on advisory boards, steering committees, safety monitoring boards and manuscript writing groups for clinical trials. CJB has additionally served as a reviewer and expert witness for regulatory authorities involved in the assessment of diabetes medicines. None of these activities is considered to unbalance the independence or objectivity of this review.
