# Supplementary material for: Pharmacological therapies for type 2 diabetes: future approaches
Source: Diabetologia. 2025 Oct 31;69(1):20–35. doi: 10.1007/s00125-025-06581-6 (PMC12686082; doi:10.1007/s00125-025-06581-6)
Supplement: Supplementary file 1 — ESM (PDF 275 KB) [file 125_2025_6581_MOESM1_ESM.pdf]

Electronic supplementary material (ESM)

Pharmacological therapies for type 2 diabetes: future approaches

Clifford J. Bailey[1]
[1]Health and Life Sciences, Aston University, Birmingham, UK

ESM Appendix 1

Why we need more glucose-lowering agents

Current management of type 2 diabetes is still far from optimal and shows considerable variations associated with national, ethnic and socio-economic factors as well as sub-types of diabetes, age, sex and co-morbidities. For example, in the USA the National Diabetes Statistics Report of 2024 noted that only 52.6% of all adults with diabetes had an HbA1c <53 mmol/mol (<7%), and HbA1c values are higher amongst Black and Hispanic peoples and people living in less wealthy areas [1, 2]. The National Diabetes Audit for England 2021-2022 recorded that only 36.3% of people with type 1 diabetes and 63.1% of people with type 2 diabetes had an HbA1c ≤58 mmol/mol (≤7.5%) [3]. Adolescents and elderly or frail individuals typically have worse than average glycaemic control based on HbA1c measurements, and studies using continuous glucose monitoring have confirmed that high glucose variability as well as high HbA1c are associated with diabetes complications [4-6].

Beyond the constraints of genetics and exposure to unalterable environmental factors there are many modifiable barriers that contribute to poor metabolic control [7]. These include inappropriate lifestyle or behaviours, development of overweight and obesity (especially visceral adiposity), inadequate adherence, social and cultural pressures, insufficient health education or knowledge, injection or tablet phobias, fear of hypoglycaemia and financial constraints. Limitations imposed by the structure of healthcare provision, scarcity of resources, insufficient clinical education, delayed diagnosis, and clinical inertia are further concerns.

Poor metabolic control is associated with earlier onset and increased severity of diabetes complications, notably retinopathic, neuropathic, nephropathic and cardiovascular diseases (the latter are often independent co-morbidities), and most people with type 2 diabetes will develop one or more complications in their lifetime [8, 9]. These complications

reduce the quality and duration of life and account for most of the direct costs of diabetes (eg at least 60% of the direct costs of diabetes in England) [10-12]. Diabetes is estimated to account for about 10% of the National Health Service (NHS) expenditure in the UK. Early intervention to re-establish and maintain near-normal blood glucose control (and to address cardiovascular risk and relevant co-morbidities) has been shown to reduce the development of complications [9, 13, 14]. Many studies have demonstrated the effectiveness of diabetes prevention programmes and large programmes of various types are underway in many communities [15].

The established links between poor metabolic control and diabetes complications sound a loud mandate for more effective interventions to improve the metabolic management of diabetes.

References for ESM Appendix 1

1. National Diabetes Statistics Report, Centers for Disease Control and Prevention 2024. https://www.cdc.gov/diabetes/php/data-research/appendix.html#cdc_report_pub_study_section_9-table-9 Accessed 9 June 2025

2. Zakaria NI, Tehranifar P, Laferrère B, Albrecht SS (2023) Racial and ethnic disparities in glycemic control among insured US adults. JAMA Netw Open 6: e2336307. doi:10.1001/jamanetworkopen.2023.36307

3. National Diabetes Audit 2021-22, Report 1: Care Processes and Treatment Targets, Overview. NHS Digital October 2023. https://digital.nhs.uk/data-and-information/publications/statistical/national-diabetes-audit/core-1-2021-22-overview#:~:text=In%20people%20with%20type%201%20diabetes%20in%20England%20the%20HbA1c,levels%20(see%20figure%201)
Accessed 9 June 2025

4. Lascar N, Brown J, Pattison H, Barnett AH, Bailey CJ, Bellary S (2018) Type 2 diabetes in adolescents and young adults. Lancet Diabetes Endocrinol 6: 69-80. doi: 10.1016/S2213-8587(17)30186-9.

5. Bellary S, Kyrou I, Brown JE, Bailey CJ (2021) Type 2 diabetes mellitus in older adults: clinical considerations and management. Nat Rev Endocrinol 17: 534-548. doi: 10.1038/s41574-021-00512-2.

6. Yapanis M, James S, Craig ME, O'Neal D, Ekinci EI (2022) Complications of diabetes and metrics of glycemic management derived from continuous glucose monitoring. J Clin Endocrinol Metab 107: e2221-e2236. doi: 10.1210/clinem/dgac034.

7. Blonde L, Aschner P, Bailey C, Ji L, Leiter LA, Matthaei S (2017) Global Partnership for Effective Diabetes Management: Gaps and barriers in the control of blood glucose in people with type 2 diabetes. Diab Vasc Dis Res 14: 172-183.

8. Zheng Y, Ley S, Hu F (2018) Global aetiology and epidemiology of type 2 diabetes mellitus and its complications. Nat Rev Endocrinol 14, 88–98. https://doi.org/10.1038/nrendo.2017.151

9. Nathan DM; DCCT/EDIC Research Group (2014) The diabetes control and complications trial/epidemiology of diabetes interventions and complications study at 30 years: overview. Diabetes Care 37: 9-16. doi: 10.2337/dc13-2112.

10. Harding JL, Pavkov ME, Magliano DJ, Shaw JE, Gregg EW (2019) Global trends in diabetes complications: a review of current evidence. Diabetologia 62: 3-16. doi: 10.1007/s00125-018-4711-2.

11. Dal Canto E, Ceriello A, Rydén L et al (2019). Diabetes as a cardiovascular risk factor: An overview of global trends of macro and micro vascular complications. Eur J Prev Cardiol 26: Suppl 2, 25-32. doi: 10.1177/2047487319878371.

12. Hex N, MacDonald R, Pocock J et al (2024) Estimation of the direct health and indirect societal costs of diabetes in the UK using a cost of illness model. Diabetic Med 41, e15326.

13. Khunti K, Zaccardi F, Amod A et al (2025) Glycaemic control is still central in the hierarchy of priorities in type 2 diabetes management. Diabetologia 68, 17–28. https://doi.org/10.1007/s00125-024-06254-w

14. Cai J, Liu J, Lu J et al (2025) Impact of time in tight range on all-cause and cardiovascular mortality in type 2 diabetes: A prospective cohort study. Diabetes Obes Metab 27: 2154-2162. doi: 10.1111/dom.16212.

15. McMullen B, Duncanson K, Collins C, MacDonald L, Wicks A (2024) Systematic review of the mechanisms influencing engagement in diabetes prevention programmes for people with pre-diabetes. Diabetic Med 41: s15323. https://doi.org/10.1111/dme.15323.

ESM Appendix 2

Limitations of existing therapies: identifying unmet needs

Table 1 of the main manuscript notes the variety of mechanisms of action of currently available glucose-lowering medications. Most advanced stages of type 2 diabetes require combinations of two or more differently acting non-insulin glucose lowering agents in order to maintain an acceptable level of glycaemic control, and addition of insulin is usually reserved until non-insulin options are inadequate. The efficacy of glucose-lowering

medication is substantially dependent on disease progression and particularly β-cell function in the face of insulin resistance. However, exogenous insulin or agents that stimulate insulin secretion irrespective of the prevailing glucose concentration (sulfonylureas and meglitinides) carry the risk of overt hypoglycaemia. This requires especial attention to dose titration, lifestyle (notably diet), self-monitoring of blood glucose and cautions over driving and physical activities. Risk of hypoglycaemia is also to be considered with use of combination therapies. Most agents are contraindicated for pregnancy and lactation, excepting insulin and the possible use of metformin in some circumstances. Other common contraindications and cautions are listed in the table below (ESM Table 1). For specific pharmacodynamic and pharmacokinetic constraints, potential drug interactions and practical concerns, readers are referred to the individual product labels (summaries of product characteristics) available at the Electronic Medicines Compendium https://www.medicines.org.uk/emc#gref (accessed 18 June 2025).

Although disease heterogeneity can considerably alter the phenotypic presentation of type 2 diabetes, underlying lesions affecting pancreatic beta-cell function and number, insulin resistance and glucotoxicity present unmet needs and potential therapeutic targets for almost all presentations. Pancreatic beta-cell defects generate a wish list of agents to restore first phase glucose-induced insulin release and proinsulin-to-insulin processing. To address the diminishing beta-cell population, further agents that reduce beta-cell apoptosis or promote beta-cell neogenesis would be highly advantageous. Research that can exploit advances in cell reprogramming, microRNAs and gene editing offers particular opportunities here.

Because insulin resistance has a detrimental effect on many physiological functions beyond nutrient metabolism, it remains an important and inadequately addressed therapeutic target. However, insulin signalling is often disrupted at more than one step along the signalling pathways, imposing rate-limiting constraints that diminish the effectiveness of individual interventions. Similarly, an intervention that directly targets a particular step in a metabolic pathway may be impeded by ‘pinch-points’ at other steps in the pathway.

Agents to regulate food intake and improve weight control offer recognised opportunities to benefit beta-cell function, insulin resistance and glucotoxicity as well as reducing risk of associated morbidities that commonly accompany type 2 diabetes. Many agents are already in development to take advantage of this approach.

ESM Table 1. Limitations of existing therapies for type 2 diabetes.

Class	Contraindications, limitations and risks
**Metformin**	Renal impairment; risk of lactic acidosis with advanced kidney disease; alcohol abuse; vitamin B12 deficiency; gastrointestinal disturbances may limit dose titration
**Sulfonylureas**	Hypoglycaemia; weight gain; durability may be reduced by hastened beta-cell exhaustion; cautions with advanced kidney or liver disease (depending on drug elimination route)
**Meglitinides**	Hypoglycaemia and weight gain are lesser risk than for sulfonylureas; caution with advanced liver disease
**DPP4 inhibitors**	Some DPP4 inhibitors require dose adjustment in advanced kidney disease
**Pioglitazone**	Weight gain; oedema (especially with insulin), heart failure; advanced liver disease; possible increased risk of bone fracture
**SGLT2 inhibitors**	Severe kidney disease/dialysis; dehydration; history of ketoacidosis; recurrent urinary infections
**Alpha-glucosidase inhibitors**	Gastrointestinal disorders (especially obstructive); advanced kidney or liver disease
**GLP-1 receptor agonists**	Gastrointestinal disorders (especially obstructive); pancreatitis; end-stage kidney disease; ketoacidosis. Cost; mostly given by injection.
**Insulin**	Hypoglycaemia; weight gain. Injection.
And in some countries	
**Pramlintide**	Gastroparesis; hypoglycaemia unawareness; adjustment of insulin and meals. Injection
**Bromocriptine**	Hypertension; cardiovascular caution if long-term use
**Colesevelam**	Gastrointestinal disorders (especially obstructive); pancreatitis; biliary disorders

ESM Appendix 3

Potential therapies undeveloped, discontinued or not completed due to unresolved issues during development

This appendix describes novel glucose-lowering mechanisms and interventions that have been discontinued or incurred unresolved issues during preclinical or clinical development but provide valuable lessons regarding potential therapeutic opportunities.

Nutraceuticals

Type 2 diabetes is frequently associated with deficiencies of intake and/or circulating concentrations of several vitamins and minerals, notably vitamins D (cholecalciferol), C (ascorbic acid), E (alpha-tocopherol), beta-carotene, B1 (thiamine), B12 (cyanocobalamine) and H (biotin), and minerals magnesium, chromium, zinc and iron [1]. Dietary supplementation to correct (but not over-correct) micronutrient deficiencies can often modestly improve glycaemic control but this is seldom a permanent disease-modifying intervention [2]. Similarly, prebiotic (non-digestible fibre) supplements can assist glycaemic management, and a substantial literature (outside of the present remit) is available regarding high fibre diets [3]. Evidence concerning probiotic (live organism) supplements is less consistent and much may depend on the pre-existing microbiome and diet, as well as the organisms within the supplements [4]. Endless claims of benefits from supplements containing antioxidants or possible anti-inflammatory factors have been difficult to validate, and although such supplements may provide modest assistance for individuals with particular deficiencies, they have not provided long-term protection against disease progression [4].

Insulin mimetics

The therapeutic potential of agents that can mimic insulin or potentiate insulin signalling has been hampered by multiple 'bottle-necks' in the signalling pathways such that the benefit of facilitating one step in the pathway is diminished by rate-limiting restrictions at distal steps [5, 6]. Regarding activation of the insulin receptor, crystal structures have shown that the interaction with insulin involves two spatially separated sites within the extracellular domain of the insulin receptor. This illustrates why the development of small molecule insulin receptor agonists has been elusive [7]. The subtle manner of the interaction between insulin and the insulin receptor is further illustrated by studies with a monoclonal antibody showing that a different binding arrangement can create a different conformational change that enables only some of the intracellular effects of insulin [8]. Nevertheless, proof of principle for a non-peptide insulin receptor agonist is demonstrated by a small molecule fungal metabolite (chaetochromin A). This interacts with the extracellular domain of the insulin receptor independently of insulin binding, initiating phosphorylation of the intracellular receptor domain which in turn activates insulin receptor substrate IRS-1 and signalling through Akt (protein kinase B) to increase glucose uptake by muscle cells. Oral administration of the compound also lowered blood glucose in diabetic mice [9]. Another fungal metabolite, demethylasterriquinone, has initiated insulin action and lowered blood glucose independently of insulin by interaction directly with the intracellular domain of the beta-subunit of the

insulin receptor [10]. Also, several small molecules have been shown to interact with the intracellular domain of the insulin receptor to increase insulin receptor signalling after receptor phosphorylation has been initiated by insulin, but none of the above has progressed for clinical evaluation [11]. It is noted that interventions to mimic insulin action must avoid over compensation, to prevent risk of 'hypos' and other adverse effects seen with hyperinsulinaemia.

Phosphatase inhibition

Inhibitors of protein tyrosine phosphatase-1B (PTP1B) can interrupt deactivation of the insulin receptor (eg. metformin inhibits PTP1B) and thereby prolong post-receptor signalling. Vanadium salts provide an interesting example because they can lower blood glucose in type 2 diabetes and the effect lasts for several days, but the narrow therapeutic window and 'off-target' effects have precluded development [12].

Post-receptor insulin signalling

Several interventions have increased insulin post-receptor signalling by inhibiting intermediates that exert negative feedbacks, such as some isoforms of protein kinase C which cause serine phosphorylation of IRS-1 to preclude signalling. Agents that boost the intracellular supply of signalling intermediates can increase insulin action: for example, methyl-chiroinositol (pinitol) increases signalling by phosphatidylinositol 3-kinase. However, non-specific effects have limited the application of this approach [13].

Adipokines

Receptor agonists and antagonists have been developed for several adipokines that provide potential opportunities to modify insulin action or appetite control, but none has yet to fulfil the requirements for an effective long-term medicine for glycaemic control in type 2 diabetes. Small molecule agonists of adiponectin receptors (AdipoR1 and AdipoR2) continue in consideration as opportunities to reduce gluconeogenesis and increase fatty acid oxidation via activation of AMPK and PPAR-alpha [14]. Adipocyte peptides that support insulin action such as apelin, omentin and visfatin, or peptides that impede insulin action such as resistin, retinol-binding protein-4, tumour necrosis factor-alpha and interleukin-6 also remain in consideration as therapeutic targets for type 2 diabetes [15]. To specifically reduce adipose tissue mass without affecting lean body mass, preclinical studies have inhibited the activin receptor-like kinase-7 (ALK7), which is predominantly expressed in adipose tissue. Gene

knock-out, a neutralising antibody or a small interfering RNA to inhibit ALK7 have increased catecholamine sensitivity, lipolysis and fat oxidation and achieved weight loss while preserving muscle [16].

Duodenal mucosal disruptors

Surgical procedures that by-pass the duodenum, or endoscopic interventions that damage the duodenal mucosa or prevent contact between the duodenal mucosa and content of the lumen have all been reported to reduce hyperglycaemia and improve weight control in type 2 diabetes [17]. The efficacy of Roux-en-Y gastric by-pass (RYGBP), mini-gastric by-pass (MGBP), and biliopancreatic diversion (BPD) is well recognised, and endoscopic procedures such as an endoluminal duodenal liner (Endobarrier) or thermal, electroporation or laser disruption of the duodenal mucosa have all improved glycaemic and weight control. This illustrates the opportunity for further consideration of intestinal peptides as templates for new glucose-lowering therapies [18].

Selective peroxisome proliferator-activated receptor modulators

Previously approved thiazolidinedione molecules have improved insulin sensitivity, but side effects including weight gain, risk of oedema and other concerns have precluded further usage in many regions. Manipulation of thiazolidinedione and non-thiazolidinedione molecules to selectively regulate the peroxisome proliferator-activated receptors (PPARγ, PPARα and PPARδ, collectively referred to as selective peroxisome proliferator-activated receptor modulators, SPPARMs) remains in consideration. However, this approach has yet to minimise unwanted side effects sufficiently to yield new glucose-lowering compounds for full development [19].

Hydroxysteroid dehydrogenase-1 inhibitors

Hypercortisolism is often a finding in individuals with poorly controlled type 2 diabetes, and inhibition of 11β-hydroxysteroid dehydrogenase-1 can reduce cortisol concentrations by preventing conversion of cortisone back to active cortisol in the liver and adipose tissue. Such inhibitors have improved insulin sensitivity, glycaemic control and weight control in type 2 diabetes patients, but compensatory increases in adrenocorticotropic hormone (ACTH) remain problematic [20].

Adenosine monophosphate-activated protein kinase activators

Activators of adenosine monophosphate-activated protein kinase (AMPK) provide a recognised approach to improve metabolic control in type 2 diabetes by enhancing glucose uptake and oxidation, decreasing gluconeogenesis, decreasing lipogenesis and increasing fatty acid oxidation. Metformin, some PPARγ agonists, alpha-lipoic acid, adiponectin receptor agonists and various analogues of AMP such as AICAR (5-amino imidazole-4-carboxamide-1-B-D-ribofuranoside) can activate AMPK. Further activators have received preclinical evaluation, but have not entered or not completed clinical assessment [21]. Increasing the action of AMPK has the added advantage of suppressing the mammalian target of rapamycin complex (mTORC) and indirectly inhibiting nuclear factor kappa-B (NF-κB), so reducing the production of proinflammatory cytokines.

Inhibitors of hepatic glucose output, glucose production and metabolism

Inhibitors of glucose 6-phosphatase and fructose 1,6 bisphosphatase, which suppress hepatic gluconeogenesis and/or glycogenolysis have shown effective blood glucose lowering activity in preclinical and clinical studies, but the avoidance of hypoglycaemia remains a particular challenge [22].

Sirtuins

Sirtuins are nicotinamide-adenine-dinucleotide-dependent histone deacetylases and ADP-ribosyltransferases. Small molecule activators of several sirtuins (notably SIRT1) have provided an epigenetic route to modify the transcription of genes that increase mitochondrial biogenesis and energy expenditure, improve body weight control, lower blood glucose and slow the progression of nephropathy and neuropathy in diabetic animal models [23]. However, the results of clinical trials with some of these activators have been mixed, and none has proceeded successfully in clinical development [24].

Bromodomain and extra-terminal (BET)) proteins exert epigenetic effects via interaction with acetylated histones, and some small molecule modulators have protected against cytokine-induced beta-cell apoptosis and improved insulin sensitivity in preclinical studies [25]. However, glucose-lowering effects suited to the treatment of type 2 diabetes have yet to emerge during clinical studies [26].

Mitophagy agents

Because the removal of ageing and damaged mitochondria (mitophagy) can reduce oxidative stress and chronic inflammation, and improve insulin action, interventions to increase

mitophagy have been considered as a treatment strategy to assist in the management of type 2 diabetes. These include activators of sirtuin 1 (SIRT1), AMPK and PGC-1alpha (eg thiazolidinediones) as considered above [27, 28].

Diacylglycerol acyltransferase regulation

Diacylglycerol acyltransferase enzymes (DGAT1/2) convert diacylglyceride to triglyceride, and inhibitors of DGAT1 have reduced lipid storage, improved insulin sensitivity and lowered blood glucose in preclinical models, but clinical studies in type 2 diabetes have yet to be successful [29].

Testosterone and selective androgen receptor modulators

Testosterone replacement therapy and some selective androgen receptor modulators (SARMs) have been helpful in improving insulin sensitivity and glycaemic control in some men with type 2 diabetes, especially hypogonadal individuals with excess visceral adiposity, but efficacy is probably not sufficient for more general glucose-lowering purposes [30].

Microbiome modulators

Although the microbiome is highly variable between individuals, at least partly due to ethnic and dietary factors, type 2 diabetes is often associated with a reduction in bacteria that produce butyrate and other short-chain fatty acids. Pre- and pro-biotic supplements (considered above) and faecal transplants to increase relevant bacteria have had mixed effects, and a reliable alteration to the microbiome that produces predictable and lasting benefits to glycaemic control has yet to emerge [31].

MicroRNAs

RNA targets to improve pancreatic beta-cell function are included in the main text. Appealing targets to improve insulin action in type 2 diabetes have been identified, but potentially effective miRNA approaches have been impeded by the difficulty of limiting action to selected tissues [32, 33].

References for ESM Appendix 3

1. Mangal DK, Shaikh N, Tolani H et al (2025) Burden of micronutrient deficiency among patients with type 2 diabetes: systematic review and meta-analysis: BMJ Nutrition,

Prevention & Health 2025; e000950.
https://nutrition.bmj.com/content/early/2025/01/03/bmjnph-2024-000950

2. Fong C, Alesi S, Mousa A et al (2022) Efficacy and safety of nutrient supplements for glycaemic control and insulin resistance in type 2 diabetes: an umbrella review and hierarchical evidence synthesis. Nutrients 14: 2295. https://doi.org/10.3390/nu14112295

3. Mao T, Huang F, Zhu X, Wei D, Chen L (2021) Effects of dietary fiber on glycemic control and insulin sensitivity in patients with type 2 diabetes: A systematic review and meta-analysis. J Functional Foods 82: 104500

4. Rondanelli M, Borromeo S, Cavioni A (2025) Therapeutic strategies to modulate gut microbial health: approaches for chronic metabolic disorder management. Metabolites 15: 127. doi: 10.3390/metabo15020127

5. Bailey CJ (2024) Future Drug Treatments for Type 2 Diabetes. In: Holt RIG, Flyvbjerg A (eds) Textbook of Diabetes, Sixth Edition. Wiley, Chichester, pp 1154-1166. https://doi.org/10.1002/9781119697473.ch79

6. Batista TM, Haide N, Kahn CR (2021) Defining the underlying defect in insulin action in type 2 diabetes. Diabetologia 64: 994–1006

7. Menting JG, Whittaker J, Margetts MB et al (2013) How insulin engages its primary binding site on the insulin receptor. Nature 493: 241–5.

8. Bhaskar V, Goldfine ID, Bedinger DH et al (2012) A fully human allosteric monoclonal antibody that activates the insulin receptor and improves glycemic control. Diabetes 61: 1263–71.

9. Qiang G, Xue S, Yang JJ (2014) Identification of a small molecular insulin receptor agonist with potent antidiabetes activity. Diabetes 63: 1394–1409.

10. Webster NJ, Park K, Pirrung MC (2003) Signaling effects of demethylasterriquinone B1, a selective insulin receptor modulator. Chem Bio Chem 4: 379–85.

11. Nankar RP, Doble M (2013) Non-peptidyl insulin mimetics as a potential antidiabetic agent. Drug Discovery Today 18: 748–55.

12. Thompson KH, Lichter J, Lebel C et al (2009) Vanadium treatment of type 2 diabetes: a view to the future. J Inorg Biochem 103: 554–8.

13. Bates SH, Jones RB, Bailey CJ (2000) Insulin-like effect of pinitol. Br J Pharmacol 130: 1944-8. doi: 10.1038/sj.bjp.0703523.

14. Begum M; Choubey M; Tirumalasetty MB et al (2023) Adiponectin: a promising target for the treatment of diabetes and its complications. Life 13: 2213. https://doi.org/10.3390/life13112213

15. Bailey CJ, Day C (2018) Treatment of type 2 diabetes: future approaches. Brit Med Bull 126: 123–137. https://doi.org/10.1093/brimed/ldy013

16. Zhao M, Okunishi K, Bu Y et al (2023) Targeting activin receptor-like kinase 7 ameliorates adiposity and associated metabolic disorders. JCI Insight 8: e161229. doi: 10.1172/jci.insight.161229.

17. Bailey CJ, Flatt PR, Conlon JM (2025) Multifunctional incretin peptides in therapies for type 2 diabetes, obesity and associated co-morbidities. Peptides 187: 171380

18. Bailey CJ, Flatt PR (2024) Duodenal enteroendocrine cells and GIP as treatment targets for obesity and type 2 diabetes. Peptides 174: 171168. doi: 10.1016/j.peptides.2024.171168.

19. Frkic RL, Richter K, Bruning JB (2021) The therapeutic potential of inhibiting PPARγ phosphorylation to treat type 2 diabetes. J Biol Chem 297: 101030. doi: 10.1016/j.jbc.2021.101030.

20. Gregory S, Hill D, Grey B et al 2020) 11β-hydroxysteroid dehydrogenase type 1 inhibitor use in human disease-a systematic review and narrative synthesis. Metabolism Clinical and Experimental 108: 154246

21. Aydin S, Tekinalp SG, Tuzcu B (2024) The role of AMP-activated protein kinase activators on energy balance and cellular metabolism in type 2 diabetes mellitus. Obesity Medicine 53: 100577

22. Kaur R, Dahiya L, Kumar M (2017) Fructose-1,6-bisphosphatase inhibitors: A new valid approach for management of type 2 diabetes mellitus. European J Med Chem 141(2B) doi: 10.1016/j.ejmech.2017.09.029

23. Dewanjee S, Vallamkondu J, Kalra RS et al (2021) The emerging role of HDACs: pathology and therapeutic targets in diabetes mellitus. Cells 10: 1340. doi: 10.3390/cells10061340.

24. Curry AM, White DS, Donu D, Cen Y (2021) Human sirtuin regulators: The "Success" stories. Front Physiol 12: 752117. doi: 10.3389/fphys.2021.752117.

25. Negi V, Lee J, Mandi V et al (2024) Bromodomain protein inhibition protects β-cells from cytokine-induced death and dysfunction via antagonism of NF-κB pathway. Cells 13: 1108. doi: 10.3390/cells13131108.

26. Wu D, Duan Q (2022) Roles of bromodomain extra terminal proteins in metabolic signaling and diseases. Pharmaceuticals 15: 1032. https://doi.org/10.3390/ph15081032

27. Shan Z, Fa WH, Tian CR, Yuan CS, Jie N (2022) Mitophagy and mitochondrial dynamics in type 2 diabetes mellitus treatment. Aging (Albany NY) 14: 2902-2919. doi: 10.18632/aging.203969.

28. Ning P, Jiang X, Yang J, Zhang J, Yang F, Cao H (2022) Mitophagy: A potential therapeutic target for insulin resistance. Front Physiol 13: 957968. doi: 10.3389/fphys.2022.957968.

29. Naik R, Obiang-Obounou BW, Kim M, Choi Y, Lee HS, Lee K (2014) Therapeutic strategies for metabolic diseases: Small-molecule diacylglycerol acyltransferase (DGAT) inhibitors. Chem Med Chem 9: 2410-24. doi: 10.1002/cmdc.201402069.

30. Wittert G, Bracken K, Robledo KP et al (2021) Testosterone treatment to prevent or revert type 2 diabetes in men enrolled in a lifestyle programme (T4DM): a randomised, double-blind, placebo-controlled, 2-year, phase 3b trial. Lancet Diabetes Endocrinol 9: 32-45.

31. Liu L, Zhang J, Cheng Y et al (2022) Gut microbiota: A new target for T2DM prevention and treatment. Front Endocrinol (Lausanne) 11: 13:958218. doi: 10.3389/fendo.2022.958218.

32. Kariuki D, Aouizerat BE, Asam K et al (2023) MicroRNA biomarkers target genes and pathways associated with type 2 diabetes. Diabetes Res Clin Pract 203:110868. doi: 10.1016/j.diabres.2023.110868.

33. Palihaderu P, Mendis B, Premarathne J (2022) Therapeutic potential of miRNAs for type 2 diabetes mellitus: an overview. Epigenet Insights 15: 25168657221130041. doi: 10.1177/25168657221130041.
