# Supplementary material for: Pharmacological therapies for type 2 diabetes: future approaches
Source: Diabetologia. 2025 Oct 31;69(1):20–35. doi: 10.1007/s00125-025-06581-6 (PMC12686082; doi:10.1007/s00125-025-06581-6)
Supplement: Supplementary file 2 — Figure slide (PPTX 297 KB) [file 125_2025_6581_MOESM2_ESM.pptx]

## Slide 1
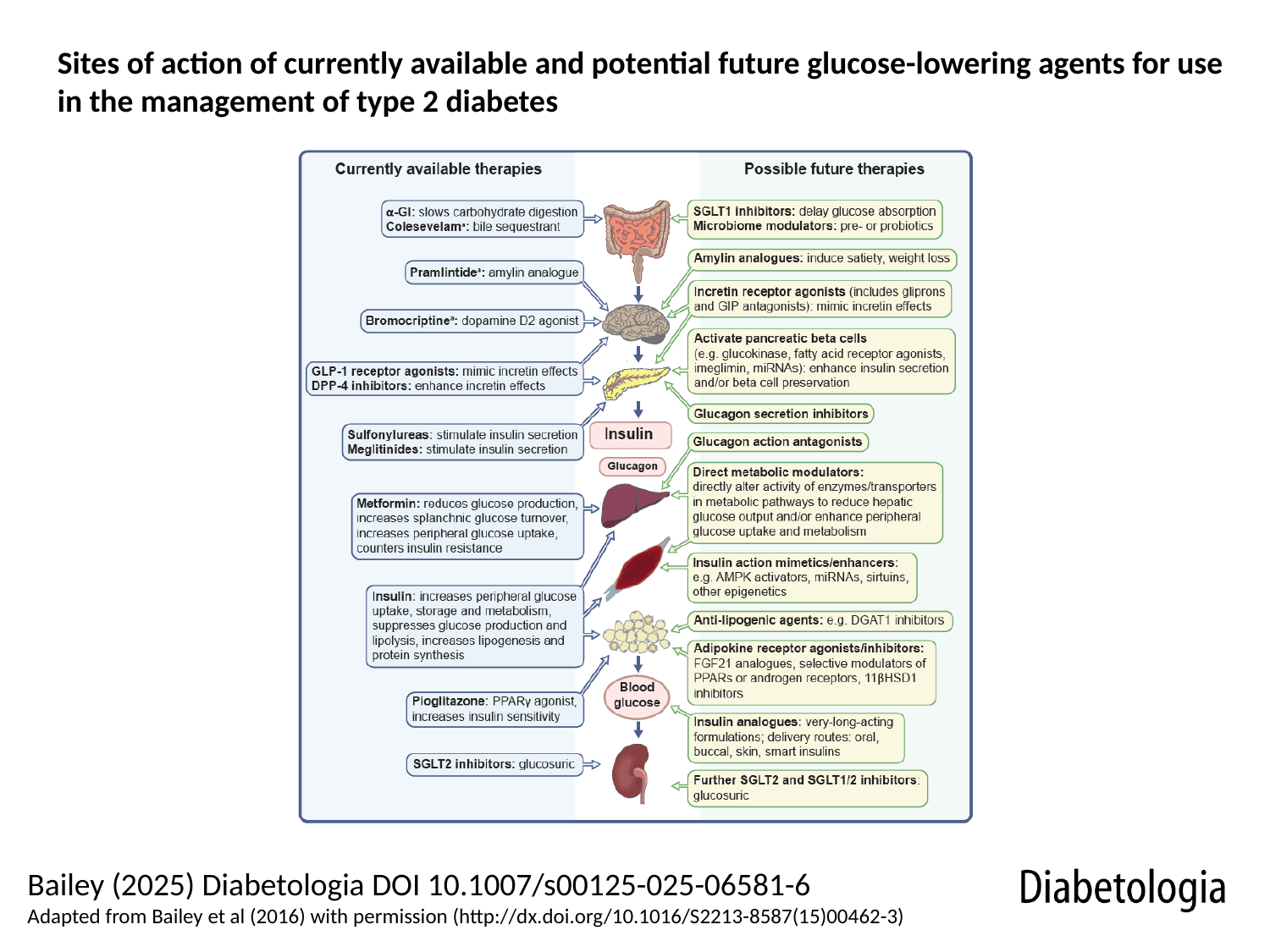

Sites of action of currently available and potential future glucose-lowering agents for use in the management of type 2 diabetes
Bailey (2025) Diabetologia DOI 10.1007/s00125-025-06581-6
Adapted from Bailey et al (2016) with permission (http://dx.doi.org/10.1016/S2213-8587(15)00462-3)
